# Invasive Candidiasis: Update and Current Challenges in the Management of This Mycosis in South America

**DOI:** 10.3390/antibiotics11070877

**Published:** 2022-06-30

**Authors:** Fernando Oscar Riera, Juan Pablo Caeiro, Sofia Carla Angiolini, Cecilia Vigezzi, Emilse Rodriguez, Paula Alejandra Icely, Claudia Elena Sotomayor

**Affiliations:** 1 Division of Infectious Diseases, Sanatorio Allende, Córdoba 5000, Argentina; fernandooriera@gmail.com; 2 Research Group of Immunology and Mycology, Córdoba 5000, Argentina; jpcaeiro3@gmail.com (J.P.C.); sangiolini@unc.edu.ar (S.C.A.); cvigezzi@fcq.unc.edu.ar (C.V.); emirodriguez@unc.edu.ar (E.R.); picely@fcq.unc.edu.ar (P.A.I.); 3 Section of Infectious Diseases, Hospital Privado Universitario de Córdoba, Córdoba 5000, Argentina; 4 Laboratory of Innate Immunity to Fungal Pathogens, Departamento de Bioquímica Clínica, Facultad de Ciencias Químicas, Universidad Nacional de Córdoba, Córdoba 5000, Argentina; 5 CIBICI-CONICET, Facultad de Ciencias Químicas, Universidad Nacional de Córdoba, Córdoba 5000, Argentina

**Keywords:** invasive candidiasis, candidemia, South America, risk factors, treatment

## Abstract

Invasive candidiasis encompassing *Candida* bloodstream infections and deep-seated candidiasis can become a persistent health problem. These infections are caused by *Candida* species and have high morbidity and mortality rates. Species distribution, access to diagnosis, treatment and mortality are different around the world. The mortality rate is high in South America (30–70%), and *Candida albicans* is the most prevalent species in this region. However, a global epidemiological shift to non-*albicans* species has been observed. In this group, *C. parapsilosis* is the species most frequently detected, followed by *C. tropicalis,* and at a slower rate, C. *glabrata,* which has also increased, in addition to the emerging *C. auris*, resistance to several drugs. This article summarizes relevant aspects of candidemia pathogenesis, such as the mechanisms of fungal invasion, immune response, and the impact of genetic defects that increase host susceptibility to developing the infection. We also discuss relevant aspects of treatment and future challenges in South America.

## 1. Introduction

Invasive candidiasis encompasses *Candida* bloodstream infection and deep-seated candidiasis [1]. These diseases are caused by *Candida* species and have high morbidity and mortality rates. Candidemia remains a significant healthcare-associated problem in several countries [2,3,4]. In South America, where most of the countries are considered low-to-middle-income nations (LMIN), the incidence of candidemia ranges from 0.74–6.0 per 1000 hospital admissions, and despite all advances in the development of new diagnostic and therapeutic tools for fungal infections, failures in infection control plus the struggle to apply appropriate treatment due to costs and delays determine that the current mortality rate achieves 30–78% [5,6].

## 2. Epidemiology

Distinct *Candida* species can cause human diseases, but most invasive infections are provoked by five pathogens: *Candida albicans*, *Candida glabrata*, *Candida tropicalis*, *Candida parapsilosis* and *Candida krusei*. It is important to keep in mind that, in agreement with recent updates in the clinical microbiology nomenclature [7], *Candida krusei* and *C. glabrata* are no longer considered members of the genus *Candida;* however, we will continue referring to them here due to their common use in the clinic. Although *C. albicans* is the most prevalent *Candida* spp. responsible for diseases, non-*albicans* candidemia caused by *C. glabrata, C. parapsilosis*, and *C. tropicalis* has also become of concern during the last decades [8]. The prevalence of non-*albicans Candida* spp. in most regions is usually determined by different factors, such as antifungal usage in the region, individual risk factors, and outbreaks involving molecular strains of *Candida* spp. that are unique to determined health-care settings [9,10,11]. In the United States and northwestern Europe, the second species most frequently found in non-outbreak settings is *C. glabrata*. This species is also more common among individuals older than 60 years and recipients of solid organ transplants [12]. In Southern Europe, India and Pakistan, *C. parapsilosis* and/or *C. tropicalis* are much more frequently encountered than *C. glabrata*. *C. krusei* is the least common of the five major *Candida* spp. [5,12].

**Figure 1:**
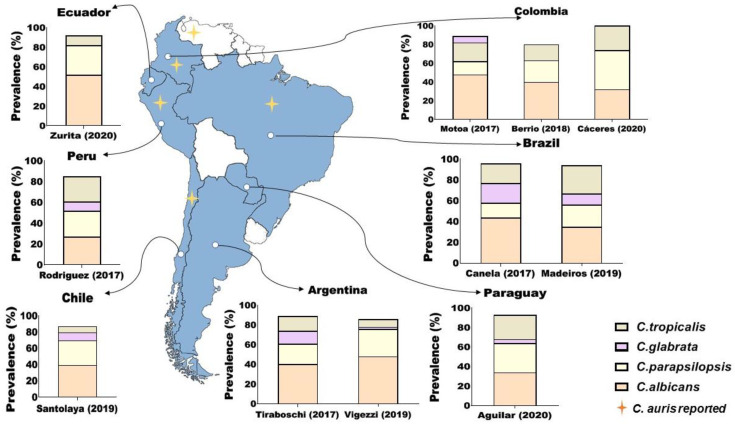
Epidemiology of candidemia in South America (2017–2022). Data about *C. auris* were recently published by the Pan American Health Organization (PAHO) [28]. Species distribution might have changed since these data were collected.

In South America, reports published during the last five years indicate that *C. albicans* remains the most prevalent species, while the most frequent non-*albicans* species is *C. parapsilosis,* followed by different species, depending on the country (Figure 1). In the central region of Argentina, our patients with systemic infections from intensive care units (ICU) had a prevalence of 48.6% for *C. albicans,* followed by 28.6% for *C. parapsilosis* [13]. These two species were the only ones isolated in patients younger than 60 years, while the elderly were also infected with *C. tropicalis* (8.7%), *C. krusei* (5.7%), and *C. glabrata* (2.3%) in a minor proportion [13]. In the eastern region of the country, Tiraboschi et al. [14] analyzed 374 episodes of candidemia, reporting a prevalence of *C. albicans:* 40.9%, *C. parapsilosis*: 21.7%, *C. tropicalis:* 15.5% and *C. glabrata*: 13.9%. In Chile, Santolaya et al. [15] provided data from 384 cases of candidemia in patients from 18 different hospitals. In this study, 35% were paediatric and 65% adult cases; the leading species were *C. albicans*: 39%, *C. parapsilosis*: 30% and *C. glabrata*: 10%, with a significant difference in the distribution of species between ages. In north-east Brazil, Madeiras et al. [16] reported a distribution of *C. albicans*: 35.3%, *C. tropicalis*: 27.4%, *C. parapsilosis*: 21.6% and *C. glabrata*: 11.8%. In northern areas of this country, Canela et al. [17] reported that *C. albicans* was the predominant species (44%), followed by a great proportion of *C*. *glabrata* (19%), *C. tropicalis* (19%), and *C. parapsilosis* (14%). In Perú, a study of 158 cases showed a low proportion of *C*. *albicans* (27.8%), with similar distribution of *C*. *parapsilosis* (25.3%), *C*. *tropicalis* (24.7%) and *C*. *glabrata* (9.5%). In Paraguay, data from 520 cases of candidemia showed prevalence values of *C. albicans*: 34.4%, *C. parapsilosis:* 30.4%, *C. tropicalis*: 25.4%, *C. glabrata*: 4.8%, and *C. krusei*: 2.1% [18]. In Colombia, three reports indicated variable proportions of *C. albicans*, *C. parapsilosis, C. tropicalis* and low or total absence of *C. glabrata* [19,20,21]. Taken together, the evaluated epidemiological data (2017–2022) of the region reaffirm the global trend in the increase of non-*albicans* species as causative agents of candidemia, with a higher prevalence of *C. parapsilosis* followed by *C. tropicalis* and an increasing trend in the frequency of *C. glabrata*, which ranged between 5–19%. With respect to the extensive revision published by Da Matta et al. [5], our collected data confirm the increase of *C. glabrata* in South America, with consequent impact on clinical management due to diminished susceptibility to azoles and echinocandins.

*C. parapsilosis* is a well-known threat for patients undergoing invasive medical interventions, as it is considered one of the leading causes of catheter-related infections and is able to produce enhanced biofilms on central venous catheters (CVCs) and other medical implants [8]. The *C. parapsilosis* complex includes three different species: *C. parapsilosis* sensu stricto, *C. metapsilosis* and *C. orthopsilosis.* In recent years, many investigations have focused on the virulence profile of these strains, host–pathogen interactions and antifungal susceptibility of cryptic species; however, even so, studies in South America remain scarce [8,13,22]. In developed areas of south-east Brazil (Sao Paulo, Rio de Janeiro and Espirito Santo), Ziccardi et al. observed a frequency of 81.1% for *C. parapsilosis* sensu stricto and 18.9% for *C. orthopsilosis* in a ten-year period (2002–2012) [23]. In Lima (Perú), a multicenter study (2009–2011) showed a prevalence of 28.1% for *C. parapsilosis* sensu lato in the absence of other cryptic species [24]. The local Candidemia Surveillance Network of Venezuela (2008–2011) reported 94.2% of *C. parapsilosis* sensu stricto, 4.6% of *C. orthopsilosis*, and 1.2% of *C. metapsilosis* [25]. We reported a frequency of 60% for *C. parapsilosis* sensu stricto and 40% for *C. orthopsilosis,* in addition to different antifungal susceptibility when comparing cryptic species of the central region of Argentina (2015–2016) [13]. *C. parapsilosis* sensu lato presented lower minimal inhibitory concentrations (MICs) for ITZ (itraconazole) compared to *C. orthopsilosis* and higher MIC values for echinocandins [6]. Similarly, Gil-Alonso et al. [22] reported that *C. metapsilosis* was the species most susceptible to echinocandins, followed by *C. orthopsilosis* and *C. parapsilosis*. Recently, in a robust study from eastern China including 884 *C. parapsilosis* species complex, Guo et al. [8] reported a frequency distribution of 86.3% for *C. parapsilosis* sensu lato, 8.1% for *C. metapsilosis*, and 5.5% for *C. orthopsilosis.* Interestingly, the resistance/non-wild-type rate of bloodstream *C. parapsilosis* sensu lato to the drugs was 3.5%, the resistance of *C. metapsilosis* to echinocandins was 7.7%, and *C. orthopsilosis* to FLZ/VRZ (fluconazole/voriconazole): 15% and to echinocandins was 5%, respectively. The MIC distribution of azoles in this study might be higher than in the Clinical and Laboratory Standards Institute CLSI M50 [4]. Further local studies providing evidence on the current distribution of cryptic species in our region and response to available antifungal drugs would be relevant for a better understanding of candidemia, its clinical care and therapeutic monitoring.

Recently, a new opportunistic *Candida* species, *C. auris,* has emerged and spread quickly to different parts of the world [12,26]. This species is of clinical concern, as most clinical isolates appear to be resistant to commonly used antifungal drugs, dramatically limiting therapeutic options and associated with high mortality rates (30–60%) [27]. The first reported outbreak of *C. auris* in America occurred in Venezuela in March 2012. Since then, different American countries have published outbreaks and isolated cases. Among them are Colombia in 2015, the United States in 2016, Panamá and Canada in 2017, Costa Rica and Chile in 2019, as well as Perú and Brazil in 2020.

The latest report of the Pan American Health Organization (PAHO) outlines South American countries with confirmed, possible and colonization cases of *C. auris* classified by year of the first finding (2012–2020) [28]. Argentina, Uruguay, Paraguay and Bolivia have not reported any cases of *C. auris* so far (Figure 1). On the other hand, using the tentative values proposed by the Centers for Disease Control and Prevention (CDC) of the United States, the South American clade presents the following percentages of resistance: FLZ (59%), amphotericin B (AmB) (11%), micafungin (9%) and 10% have multidrug resistance (MDR) [28].

## 3. Risk Factors

**Figure 2:**
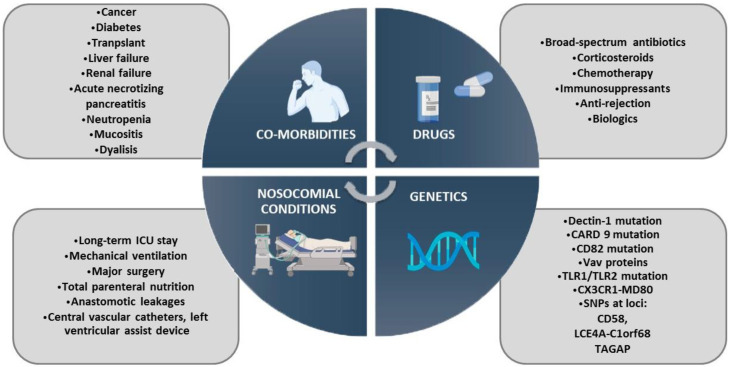
Risk Factors Predisposing to Invasive Candidiasis, categorized into four Groups: Co-Morbidities, Nosocomial Conditions, Drugs and Human Genetic Factors.

Four leading conditions predispose an invasive human infection (Figure 2) [1,12,29]. The main factors are related to hospitalization in ICUs, in addition to the usual clinical complexity of seriously ill patients, despite advances in intensive care medicine. There is no doubt that extended stay in ICUs as well as the use of mechanical supports, parenteral nutrition and CVCs are relevant factors associated with difficult eradication of the biofilms produced by many *Candida* strains. Moreover, prolonged and/or repeated use of drugs that favor yeast overgrowth and invasion by distinct mechanisms also has a deep impact. For example, the use of broad-spectrum antibiotics that break the delicate balance between resident microbiota and yeasts at the intestinal level favors the growth of *Candida* species [30,31,32]. Other drugs that downregulate the function of immune cells involved in fungal control, such as corticosteroids, chemotherapeutic agents and immunosuppressive drugs, increase the risk of fungal infections as well [33]. Many comorbidities, such as cancer, chronic diseases, neutropenia, transplants, mucositis, etc., in addition to individual host factors are associated with a greater predisposition to develop disseminated and deep infections. A recent systemic review and meta-analysis showed that broad-spectrum antimicrobials, blood transfusions, *Candida* colonization, CVCs and total parenteral nutrition were associated with the maximum risk of invasive candidiasis [34].

With the emergence of SARS-CoV-2, coinfection with *Candida* spp. in hospitalized patients with COVID-19 began to be monitored. COVID-19-associated candidiasis (CAC) involving superficial and invasive forms of infection has been explored in many countries and the reported frequency is variable. For example, in Europe, Spain has reported rates of 0.7% (7/989) [35], Italy: 8% (3/43) [36], and the UK: 12.6% (17/135) [37]. In Asia, studies from India reported 272.5% (15/596) [38], Iran 5% (53/ 1059) [39], and China reported the highest percentage: 23.5% (4/17) [40]. The presence of CAC has also been evaluated in many countries of South America, for example in Brazil, where the study performed by Camargo Martins et al. [41] reported a frequency of 4/716, being *C. albicans* the most frequently species isolates [39,42,43]. In Brazil, Nucci et al. reported several cases of *C. albicans* and non-albicans species, with a major proportion of this last type (Nucci, 2020). The general consensus indicates that the risk factors previously described, such as prolonged ICU stay, central venous catheters, and use of broad-spectrum antibiotics, may constitute the main reasons for candidemia in COVID-19-infected patients [41,42,44,45].

During the last decade, due to advances in the knowledge of human antifungal immunity and the development of next-generation techniques as part of personalized medicine, a new chapter on risk factors has been added. The description of the Dectin-1 receptor and its adapter CARD9 was a milestone in the understanding of candidiasis pathophysiology. In the clustering of Dectin-1 receptors, variants in CD82 are associated with both a risk of candidemia and decreased cytokine production upon stimulation with fungal ligands [46]. Different loss-of-function mutations in CARD9 have been related to autosomal recessive inheritance of susceptibility to invasive infections by *Candida* spp. [47]. In relation to the TLR family, Plantinga et al. proposed a mechanism for susceptibility involving the recognition of *Candida* by TLR1/TLR2 heterodimers [48]. Another example implicates a defect in the activation signaling in the response to *Candida* spp. Smeekens et al. [49] studied the downstream signaling of type I interferon and reported that STAT1 polymorphisms are associated with candidemia. Furthermore, faults associated with dysfunction in the effector mechanism involved in the control of fungal growth, especially in the ability of neutrophils to kill yeasts, predispose patients to invasive diseases. Other defects in specific adaptive immunity and human ancestry have been identified [50,51]. Due to recent advances in personalized medicine through genome-wide association studies (GWAS) [52], it would be crucial to study the genomic ancestry composed of all the different human races that inhabit our region. The understanding of immune deficiencies that underlie diseases that are still considered “idiopathic” and polygenic mechanisms of candidiasis susceptibility would provide valuable insights for clinical diagnosis and more adequate interventions in times of personalized medicine.

## 4. Pathogenesis of Invasive Candidiasis

**Figure 3:**
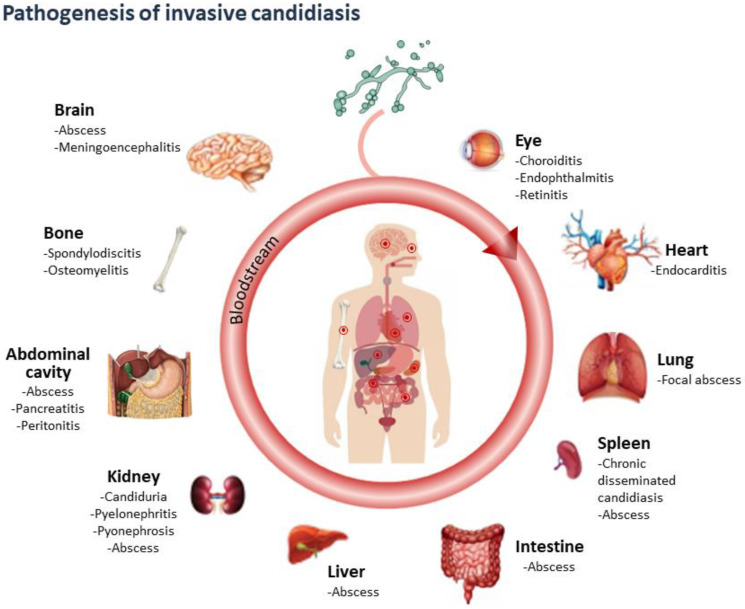
Invasive Candidiasis: Organs Susceptible of Infection by *Candida* spp. and Resulting Diseases. The names of diseases agree with those described by Pappas et al. [12].

*C. albicans* is a particular pathogen because it is uniquely adapted to the human host and can behave as either a harmless commensal or opportunistic pathogen [53,54]. In normal conditions, it colonizes the skin and mucosa of the gastrointestinal tract (GIT), oral cavity, and reproductive tract in a high percentage of healthy individuals [55,56], as regular components of the human commensal microbiota. Its growth is strictly controlled by the host immune system and the regulatory mechanisms provided by the normal microbiota [31]. Changes in this balance determine overgrowth of the fungus [57] on surfaces where it is normally found, in addition to severe systemic infections with the involvement of several organs. This fungus can colonize practically any tissue and clinical manifestations are diverse depending on the affected site (Figure 3); however, the preference for a particular organ depends largely on the route of infection, intrinsic characteristics of the pathogen and underlying conditions of each patient.

The intestinal population of *C. albicans* is considered the main source of endogenous infection; its crossing through the intestinal barrier is the beginning of spreading, causing a lethal bloodstream infection (candidemia), which can lead to invasive disease. Data collected in postmortem studies over a 12-year period demonstrated that 54% of the patients with positive blood cultures for *C. albicans* had invasive candidiasis characterized by deep-organ lesions in kidneys (80%), brain (52%), and heart (48%), showing that any tissue is susceptible to infection [58]. Lewis et al. [59] analyzed autopsies of cancer patients collected over 20 years and reported an increase in the incidence of systemic candidiasis as well as the presence of *C. albicans* in different anatomical sites, such as lung (79%), GIT (35%), kidney (34%), liver (20%) and spleen (19%). Patients with persistent neutropenia are more susceptible to renal and cardiac invasion.

### Host–Pathogen Interaction: Virulence Factors and Host Response

The first step in the invasion of epithelial barriers is mediated by multiple surface moieties, collectively called adhesins, which are expressed in the fungus and involved in its attachment to the host cells. These epithelial-yeast interactions stimulate morphogenetic changes in the fungus, exposing several hyphal-associated adhesins that further promote adherence. These well-studied proteins include the Als family, particularly Als3, and hyphal wall protein 1 (Hwp1) [60,61,62]. Following adherence, epithelial invasion can occur through induced endocytosis, which involves fungal invasins, such as Als3 and Ssa1 [61,63], or active penetration. In the first type of invasion, *C. albicans* prevents endolysosomal maturation and continues to grow. Intracellular hyphal extension depends on EED1 (Epithelial Escape and Dissemination 1) expression [64] and continued fungal elongation results in the piercing of epithelial cells and subsequent spreading.

On the other hand, active penetration requires viable fungi and results from hyphal extension and invasion between or through epithelial cells. In this process, many hydrolytic enzymes, such as aspartic proteinases (Saps) (especially Sap3), lipases and phospholipases, are involved in fungal-induced epithelial damage and passage through the intestinal epithelium [65]. Moreover, the cytolytic peptide toxin candidalysin has recently been recognized as participating in the damage of host enterocytes [29].

Nosocomial infections are closely associated with a relevant attribute of virulence of many *Candida* species; their ability to form biofilms confers an intrinsic resistance against various antifungal drugs and mechanisms of immune reaction [20]. The biofilms grow attached to medical devices or host tissues [18] and in addition to their difficult eradication, the final phase involves dispersion of non-adherent cells, which results in the initiation of newer biofilms and further dissemination to other tissues [52,53]. Atiencia-Carrera et al. [66] reported one extensive meta-analysis of 214 studies about the rates, types and antifungal resistance of *Candida* biofilms among hospitalized patients between 1995 and 2020. Data regarding mortality rates and geographical locations were also included. The majority of these studies belonged to Europe and Asia; only a few investigations from South America could be included, since many of them did not fulfil the inclusion criteria. The limitations of analysis heterogeneity, correlation between mortality and each type of biofilm, antifungal resistance and lack of sufficient published data hinder a conclusion. Nonetheless, early detection of biofilms and better characterization of *Candida* spp. would be valuable to diminish mortality among patients.

The concept of molecular pattern recognition through innate immunity receptors (PRRs) revolutionized our understanding of immunology and the immune response to infection. Different families of PRRs are involved in the recognition of *Candida* pathogen-associated molecular patterns (PAMPs); these are TLRs (Toll-like receptors), CLRs (C-type lectin receptors), NODs (NOD-like receptors), and RIGs (retinoic acid gene receptors). CLRs constitute a heterogeneous superfamily of transmembrane and soluble receptors, which contain conserved lectin-like domains that recognize carbohydrate polymers (mannans, glucans and chitin) found in the fungal-cell wall [67,68]. These are the best-characterized PRRs of the response to fungal pathogens and are required for recognition, phagocytosis, induction of antimicrobial effector mechanisms, and inflammatory mediators, as well as direction and modulation of adaptive immunity, including Th1 and Th17 responses [69].

β-glucan receptor Dectin-1 is the most studied CLR. Ferwerda et al. [67] identified a Y238X polymorphism encoding the human receptor Dectin-1, which results in little or no expression of the molecule. In vitro assays performed with peripheral blood mononuclear cells from these individuals demonstrated failure in the recognition of β-glucans of the *C. albicans* wall, resulting in decreased release of cytokines, mainly IL-17, TNF and IL-6 [67]. Plantinga et al. [48] studied the incidence of disseminated candidiasis in bone marrow transplant recipients with this polymorphism and reported that this mutation predisposes them to the development of mucocutaneous candidiasis but not invasive infection. However, a previous study in individuals under intensive treatment for hematological malignancies that presented with this mutation reported higher susceptibility to systemic invasion by *C. albicans* [70]. Although the presence of the Y238X polymorphism is not considered a predisposing factor for systemic candidiasis, it may constitute a high-risk factor in critically ill patients when infection is established due to medical interventions (Figure 2) [67]. 

Many antifungal responses depend exclusively on the activation of Syk kinase and CARD9, molecules involved downstream in CLR receptor signaling. The crucial role of CARD9 in the antifungal response is evidenced in humans and animals with a deficiency of this protein (*Card9-/-*), which shows increased susceptibility to *C. albicans*. Human CARD9 deficiency and its link with spontaneous development and severe cases of fungal infections were first described by Glocker et al. in 2009 [71] in a large Iranian consanguineous family consisting of 43 members. The Q295X mutation was associated with the occurrence of multiple cases of chronic mucocutaneous candidiasis and increased susceptibility to disseminated and cerebral forms of mycosis [72]. Other CARD9 polymorphisms have recently been reported [46]. Drummond et al. [73] observed that in patients with CARD9 deficiency, the defective recruitment of neutrophils to the brain contributed to an increased fungal burden in the central nervous system (CNS). Similar results were obtained in *Card9-/-* animal models of candidemia. Whibley et al. [74] reported that in mice infected with *C. tropicalis,* the poor expression of TNF in the absence of CARD9 led to uncontrolled fungal growth in the kidneys, spleen, liver and brain, suggesting a species-independent mechanism.

Phagocytic cells with candidacidal activity play a key role in the response to *Candida*. Circulating cells such as neutrophils and resident macrophages act as effector cells in the control of fungal load through the production of metabolites such as H_2_O_2_, superoxide anion, nitric oxide (NO) and peroxide nitrites, which have a more powerful candidacidal activity compared to their progenitors [47,54]. Among the cell populations of innate immunity, neutrophils are considered essential during the early response to *C. albicans*. Their absence or failure of activation/recruitment mechanisms lead to increased susceptibility to invasive fungal infections, reduced control of fungal growth and accelerated mortality, both in human and animal models [75,76]. These cells are rapidly recruited to the site of infection, contributing to fungal control by phagocytosis, respiratory burst, degranulation, release of pro- and anti-inflammatory cytokines, and release of extracellular traps (NETs) [68,75,77]. They are particularly involved in the onset of infection during the first 24–48 h, where an effective immune response is crucial to prevent disease progression [30,68,78]. However, its activation needs to be strictly controlled to avoid severe tissue lesions and immunopathological processes, with a detriment in the functioning of the organs involved.

In recent years, the role of monocytes (Mo) and tissue-resident macrophages (MΦ) has been explored [79]. In these cells, polarization in the metabolism of L-arginine is associated with two types of response; while the M1 profile is associated with host protection, M2 favors fungus resistance, making the M1 vs. M2 balance a relevant event in the outcome of *C. albicans* infection. Interestingly, we demonstrated that *C. albicans* induces strong activation of the arginase pathway and a significant reduction of NO production in human Mo, showing that the metabolic balance favors the M2 profile after fungal contact [56]. Wagener et al. [80] reported that chitin exposure during *C. albicans*–macrophage interaction is a strong inductor of arginase-1 activity in human MΦ. This observation, in addition to our results in human Mo, reveals the importance of the shifts of classically activated Mo/MΦ towards an alternative activated phenotype during their first interaction with the pathogen, in addition to the fungus strategies to guarantee its own survival. The molecular mechanisms that govern the interaction between hosts and *Candida* species need to be further investigated for a better understanding of the pathogenesis of this infection and the development of more effective therapeutic tools.

## 5. Treatment

Early identification of candidemia and treatment with appropriate antifungal drugs certainly reduce morbidity and mortality [81,82]. Because of the increasing frequency of infections caused by non-*albicans Candida* [83], increasing levels of FLZ resistance [84], and evidence that echinocandins are more effective than FLZ [85], the current international guidelines strongly recommend echinocandins instead of FLZ as initial treatment for candidemia in all adults and consider FLZ an acceptable initial therapy in non-critical patients and those unlikely to have an FLZ-resistant *Candida* species [82,86,87]. In addition, the Infectious Diseases Society of America (IDSA) guidelines [88] strongly recommend performing FLZ antifungal susceptibility testing (AFST) for all bloodstream *Candida* isolates because of possible resistance to this antifungal drug. Further recommendations propose echinocandin AFST for *C. glabrata* and *C. parapsilosis* bloodstream isolates due to emerging resistance in these species [82,86,87]. Although current guidelines recommend therapy with FLZ AFST, not all patients are tested due to a lack of supplies in regional laboratories [86]. For all bloodstream *Candida* isolates, nearly one-half of patients did not undergo testing for FLZ susceptibility [89]. Compared with FLZ AFST, echinocandin AFST is used less frequently, which is predictable because echinocandin AFST is not recommended for all bloodstream isolates [89].

Since detection and identification of *Candida* species generally takes at least 2–4 days after blood collection and culture and given that access to AFST may be limited or delayed, the initial antifungal therapy is usually selected based on local epidemiology, antifungal resistance patterns, and individual patient factors [90]. The regular practice is to initiate treatment with an echinocandin followed by de-escalation to azoles (FLZ, voriconazole-VCZ or isavuconazole-ISA) after clinical improvement. AFST results, if available, may be used to support decisions on step-down treatment and to identify instances in which therapy may be ineffective. Many patients do not receive treatment, which significantly increases mortality. The reasons for not receiving antifungal drugs include death or discharge before culture results are available. It is recommended that all patients with candidemia be assisted by infectious disease specialists for outcome improvement [91].

Brazilian guidelines for the management of candidiasis [92] summarize the best therapeutic strategies for patients with hematogenous candidiasis. The following aspects should be considered:

- *Presence of infectious complications in organs*: The occurrence of endophthalmitis, osteomyelitis, endocarditis and chronic disseminated candidiasis are good examples of clinical conditions for which antifungal therapy should be extended up to 1–6 months. If prolonged therapy is needed, oral drugs should be chosen.
- *Severity of the clinical presentation*: This issue is controversial; fungicidal drugs are usually selected for initial treatment in patients with organ failure, and FLZ is generally saved for a second event after the initial clinical response and identification of the *Candida* species.
- *Determination of Candida species*: Non-*albicans* species may exhibit lower susceptibility to FLZ, requiring dose adjustment or a therapeutic switch.
- *Risk of renal toxicity when using conventional Amp B*: The occurrence of acute renal failure in patients with renal dysfunction in ICUs, elderly patients, and those receiving other nephrotoxic drugs.
- *Previous exposure to antifungal prophylaxis regimens and/or empirical therapy*: In the case of breakthrough infections in patients exposed to the determined antifungal agent, a change in therapeutic group is indicated until the *Candida* species and its susceptibility profile are determined.
- *Central venous catheter*: The clinical management of this aspect must be discussed, considering the individual conditions of the patients.
- *Surgical removal of the infectious focus*: Cases of osteomyelitis and endocarditis are good examples of clinical situations in which surgical cleaning (or valve replacement) should be considered.

### 5.1. Candida Sepsis

**Figure 4:**
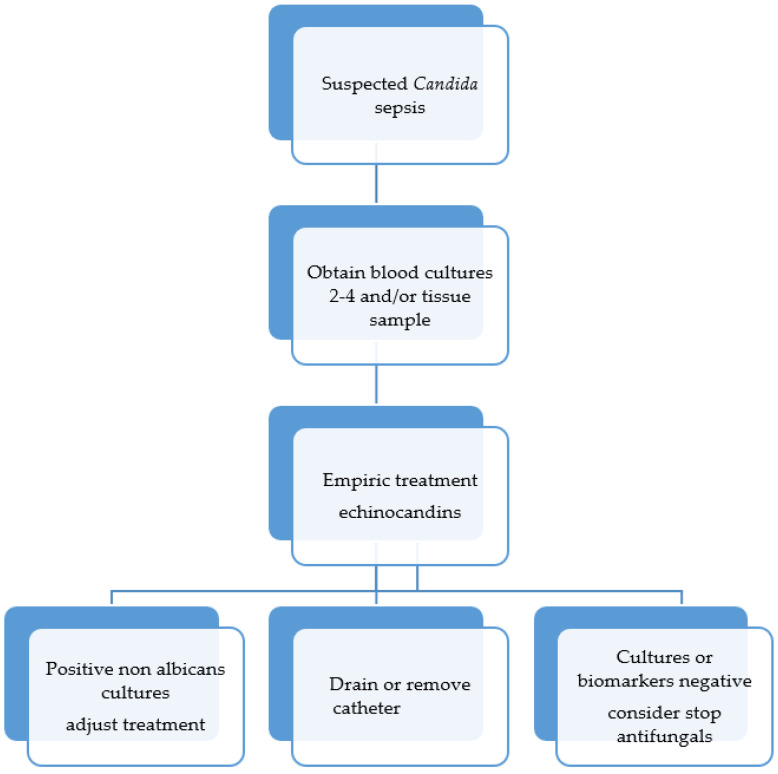
Algorithm for management of *Candida* sepsis adapted from Keighley et al. and Thompson et al. [94,95].

Fungal sepsis is frequently detected in critically ill, non-neutropenic patients. It may present as severe sepsis, septic shock, and multiple-organ failure, similar to bacterial infection [93]. Clinical management is still challenging, mainly because of the difficulties in establishing a final diagnosis. Too often, the presentation of *Candida* sepsis is very similar to sepsis of other origins, which makes it very difficult to diagnose. Half of the time, the blood cultures are negative and appropriate sterility for sampling deep-seated candidiasis may be difficult to attain. Most experts recommend empirical treatment for suspected cases and patients hospitalized in ICUs for more than 7 days with fever and hemodynamic instability and high scores in the clinical prediction rules [94]. Figure 4 shows the suggested algorithm for care.

### 5.2. Ocular Candidiasis

Candidemia may be associated with ocular involvement, chorioretinitis and/or endophthalmitis. Between 1–20% of patients suffer from this condition, sometimes without symptoms. It is recommended that patients with candidemia undergo a fundoscopic examination to evaluate the extent of their involvement. In these cases, treatment for candidemia should be maintained for up to 4–6 weeks and FLZ is the drug of choice in the absence of resistance.

### 5.3. CNS Candidiasis

In the brain, *C. albicans* produces different clinical manifestations, including meningitis, micro and macro abscesses, and vascular complications [96,97]. Meningitis is frequent in AIDS patients with low average CD4+ T lymphocytes [98]. Neurosurgical procedures can also induce *Candida* meningitis and the severity of infection correlates with the extent of the inoculum [96]. Immunosuppressed patients with candidemia and CNS invasion may develop cerebral micro abscesses, which constitute a serious complication [69]. It is also more frequent in premature infants with low birth weight. Fever, meningismus, high cerebrospinal fluid pressure, and localized neurological signs are often present. The main obstacle to treatment is the blood–brain barrier and its selective permeability to certain substances. Liposomal AmB and FLZ are recommended antifungal drugs in these cases.

Table 1 summarizes the antifungal drugs used for the clinical management of invasive candidiasis, categorized as primary and alternative, including recently introduced new drugs [98,99].

An emerging problem is the detection of antifungal resistance. Recently, Rodrigues et al. [100] reported the emergence of *C. glabrata* in southeast areas of Brazil, in addition to a significant number of strains associated with high MICs to FCZ (28.6%) and VCZ (28.6%). In addition, the presence of *C. haemulonii*, a multidrug-resistant species, was identified [100]. In Colombia, a five-year surveillance study (2016–2020) reported an increase in *C. auris* cases [101] in 379 isolates using CDC breakpoints for resistance. The author reported that 35% of resistance for FZL, 33% for AmB, 0.3% for anidulafungin and 12% were multidrug resistant. These results showed the relevance of accurate identification in the proper management of these patients.

## 6. New Antifungal Drugs

The development of effective antifungals is a big challenge because fungi and human cells are both eukaryotes and for this reason, compounds that are toxic to fungi would be probably harmful to humans too. Innovation has been slow in the antifungal field since the first echinocandins were approved barely 20 years ago. However, several companies have been working hard to develop new therapeutic options, such as these promising drugs. Ibrexafungerp, which is a novel antifungal drug from enfumafungin, is a 1,3-glucan synthase inhibitor that can be administered intravenously or by oral route and is not affected by FKS mutations. In addition, it is effective against resistant species of *Candida* [102]. Another drug, rezafungin, is a novel echinocandin with a longer half-life and can be administered once a week. Ostesaconazole is a novel tetrazole that inhibits lanosterol demethylase and Fosmanogepix is a new type of antifungal agent that inhibits Gtw1, an enzyme that traffics and anchors mannoproteins [103].

## 7. Challenges

Most South American countries constitute LMIN; in this context, three challenges should be considered: (1) rapid diagnosis of invasive candidiasis with point of care in inexpensive biomarkers, (2) new antifungal agents that are effective, affordable and available, and (3) whole genome sequencing for antifungal stewardship in real time. The most important challenge is to achieve an early diagnosis so that all patients have access to antifungal treatment. According to reported dada in Latin America, antifungal treatment is administered in 85.4% of episodes, at a median of two days after candidemia diagnosis [4,44].

## 8. Conclusions

Invasive candidiasis is a severe disease with a high incidence and mortality rate in South America. Based on epidemiological studies, it can be observed that the incidence of invasive candidiasis has increased and non-albicans species, especially *C. parapsilosis* and *C. tropicalis*, have become more frequent; although at a slower rate, cases of *C. glabrata* have also increased. As in other parts of the world, *C. auris* has emerged and the list of countries with confirmed, possible and colonization cases continues to increase. The advancements in the knowledge of human antifungal immunity and GWAS studies underlie “idiopathic” disease and the polygenic mechanism of candidiasis susceptibility; moreover, they open new routes for diagnosis and adequate interventions in times of personalized medicine. The current challenges include achieving a multimodal strategy for early identification, surveillance and notification of cases, in addition to implementing strict measures for infection control and appropriate antifungal treatment. All these issues require training, networking and better access to diagnostic tests and essential treatments with convergence of different disciplinary areas (basic and clinical research, diagnostic methods, drug design, etc.) to reduce mortality and allow effective treatment.

## Figures and Tables

**Table 1:** Suggested Drugs for Treatment of Invasive Candidiasis [88].

Candidemia Characteristic		Treatments	
	Primary	Alternative	New Drugs
**Non-Neutropenic patients**	Caspofungin Anidulafungin Micafungin	LF AmB Fluconazole * Isavuconazole Voriconazole	Ibrexafungerp Rezafungin Osteaconazole Fosmanogepix
**Neutropenic Patients**	Caspofungin Anidulafungin Micafungin	AmB Liposomal Fluconazole * Isavuconazole Voriconazole	
**Ocular Compromise +**	Fluconazole Voriconazole	AmB Liposomal	
**CNS Compromise +**	AmB Liposomal	Fluconazole	

Amphotericin B (AmB); Lipid formulation (LF); Central Nervous System (CNS) * Use in stable patients without prior use of azoles; + 6 weeks of treatment.
